# Preservation of stable isotope niche dynamics in squamate museum specimens

**DOI:** 10.1111/1365-2656.70212

**Published:** 2026-01-19

**Authors:** Maggie R. Grundler, Erica Bree Rosenblum

**Affiliations:** ^1^ Department of Environmental Science, Policy, and Management University of California Berkeley California USA; ^2^ Museum of Vertebrate Zoology University of California Berkeley California USA

**Keywords:** museum collections, niche evolution, squamates, stable isotopes

## Abstract

1. Natural history museums are invaluable resources for large‐scale ecological and evolutionary studies, but certain ecological traits can be challenging to recover, particularly from fluid‐preserved specimens. Stable isotope analysis is an elegant method for reconstructing the dietary niche over integrated timescales, and recovering this information from museum specimens can provide a critical axis of ecological information for studies of population dynamics through time and space. However, isotope ratios of tissues are known to be altered by extended contact with formalin and ethanol.
2. Here, we assess whether intra‐ and interspecific variation in isotopic signature, which represent critical data used to assess metrics of niche diversity, can be reliably recovered following fluid preservation. We use a broad taxonomic distribution of squamates to compare niche metrics prior to and 8 weeks following a standard museum preservation process
3. We could not recover intraindividual metrics of niche diversity but found that between‐individual variation was not significantly altered, allowing for the reconstruction of community niche characteristics
4. We present an example isotopic analysis from museum specimens representing generalist and specialist *Thamnophis* garter snake populations that aligns with empirical estimates of niche width
5. We also present several additional analyses on tissue‐specific effects, delipification and buffer storage, with useful insights for field collection and downstream analysis decisions.

## Graphical abstract

This paper presents the first research on the effects of fluid specimen preservation in a community context, demonstrating the recovery of metrics of niche dynamics across a diverse array of squamate taxa.

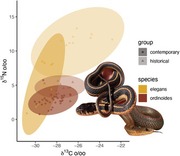

## 1 INTRODUCTION

Fluid‐preserved museum specimens represent an enormous wealth of ecological and evolutionary information. Centuries of genomic and phenotypic data can be extracted from museum collections, but some ecological traits can be difficult to recover. Dietary ecology is a particularly elusive trait to associate with individual specimens; while some animals are preserved with stomach contents, certain groups of organisms such as snakes are rarely preserved with undigested prey (and even perfectly intact stomach contents represent only a snapshot of an organism's dietary ecology; Bearhop et al., 2004). Stable isotope analysis of field samples offers an elegant solution to this issue. Because organisms assimilate isotopes from their food items, stable isotope analysis of tissues with different metabolic turnover rates provides a metric of individual dietary niche breadth by recording both short‐ and long‐term dietary habits.

While stable isotope analysis has become a commonly used method in field ecology, its application to museum specimens presents unique challenges. Formalin fixation and ethanol storage are known to impact the stable isotope ratios of animal tissues, and effects vary between tissues and taxa (Bugoni et al., 2008; Kelly et al., 2006; Sarakinos et al., 2002). While existing studies account for preservation effects in fish (Durante et al., 2020), birds (Bugoni et al., 2008) and mammals (Taylor et al., 2022), no existing studies, to our knowledge, examine preservation effects in squamate reptiles, a broad taxonomic group displaying multiple extraordinary instances of dietary diversification (Cooper & Vitt, 2002; Grundler & Rabosky, 2021). Furthermore, while prior research has focused on developing a mathematical correction of stable isotope ratios, less is known concerning the effect of specimen preservation on isotopic variance within and between individuals—in other words, the raw information used in reconstructing dietary niche dynamics. Two tissues with differing metabolic turnover rates are often used in stable isotope analysis to quantify individual specialization (Arnoldi et al., 2024; Bond et al., 2016; Petta et al., 2020), but whether this ecological signature remains following preservation has not been tested. Additionally, existing studies assess effects within individuals of a single or few species, but it remains unclear whether studies requiring a community context may reliably compare multiple preserved taxa, or whether noise introduced by fixation or from physiological differences among species obscures ecological patterns present before preservation.

As a foundational concept in evolutionary ecology, the niche is a key component of theories concerning biodiversity, including but not limited to community assembly, biogeographic transitions, phenotypic evolution and speciation (Costa‐Pereira et al., 2019; Davis et al., 2012; Egan et al., 2018). A growing body of work emphasizes the ecological and evolutionary consequences of individual variation (Bolnick et al., 2003, 2011; Costa‐Pereira et al., 2019), and the ability to retrieve relevant metrics such as individual niche breadth from museum specimens would broaden our understanding of macroecological and eco‐evolutionary dynamics. In quantifying the dietary niche, researchers often use Roughgarden's mathematical framework relating the within‐individual component, between‐individual component and total population niche width (Roughgarden, 1972, 1974). Individual specialization in this context is the result of the relative width of the individual niche compared to the total, and in isotopic space can be translated into Euclidean distances or ellipse‐based metrics of variation that rely on the assumption of a multivariate normal distribution (Bolnick et al., 2003; Jackson et al., 2011). Such assumptions may be violated if preservation differentially affects variance among individuals or tissues of different taxa.

If within‐ and between‐individual variation remain biologically meaningful following specimen preservation, researchers can more easily probe the environmental and temporal contexts of shifts in ecological community dynamics. For example, intra‐ and interindividual metrics of variation are critical when quantifying population‐ or species‐level niche breadth because they provide information on whether populations or species with wider niche breadths are composed of generalists (wide individual breadth and narrow between‐individual breadth) or individual specialists utilizing different resources (narrow individual breadth and wide between‐individual breadth). These data can support macroecological studies such as those querying patterns of niche breadth alongside range shifts, phylogenetic distributions of body size or habitat transitions with changing climate (Costa et al., 2008; Lancaster, 2022; Rather et al., 2021; Yvon‐Durocher et al., 2008). Just as museum specimens have been critical in detecting changes in morphology coincident with patterns of sympatry (Huey et al., 1974) and climate change (MacLean et al., 2019), or documenting shifting genotype frequencies through space and time (Pearse et al., 2011), isotope ratios from specimens could be used to reconstruct niche evolution in similar contexts. Determining whether the preservation process affects an organism's signature of niche breadth will therefore have important consequences for researchers looking to leverage museum specimens in evolutionary studies of dietary ecology.

To approach this question, we compared niche metrics calculated from stable isotope analysis of fresh tissue samples from a wide taxonomic sampling of squamates and from tissues of the same individuals at different time points throughout a standard museum preservation process. Because different tissues metabolize at different rates, comparing isotope ratios between slow and fast‐turnover tissues can provide an estimate of individual dietary use over time. We therefore sampled both muscle (slow metabolic turnover) and liver (fast metabolic turnover) for each individual to assess whether metrics of individual variation are affected by preservation. Existing studies show that formalin fixation causes a slight depletion of carbon isotope ratios, while ethanol causes a slight enrichment; and that both formalin fixation and ethanol preservation cause a minor enrichment of or no effect on nitrogen isotope ratios (Bugoni et al., 2008; Edwards et al., 2002; Kaehler & Pakhomov, 2001; Sarakinos et al., 2002). Further, isotope effects that do appear as a result of the preservation process are smaller than expected biological variation (Edwards et al., 2002). We therefore predicted that variation in carbon and nitrogen stable isotope signatures within and between individuals would not be significantly affected by the specimen preservation process.

We also include several comparisons related to methodological decisions in field and laboratory work for stable isotope analysis, including the difference in isotope ratios between pure muscle and whole tail samples; the effect of delipification on muscle and liver tissues; and the effect of the commonly used storage buffer RNAlater. We hope these additional analyses are informative for future research and sampling design.

## 2 METHODS

### 2.1 Specimen preservation and sampling

Specimens from the East Bay Vivarium (EBV) in Berkeley, California, were deposited at the Museum of Vertebrate Zoology at the University of California, Berkeley (because this study worked solely with deceased animals, no institutional ethical approval was required). Thirty of these individuals deposited between 2013 and 2014 were selected, including 19 lizards (10 species) and 11 snakes (7 species). Animals housed at the EBV are fed a regimented diet depending on preferences and feeding frequency in the wild, as well as the animal's age. Diets ranged from one to four bulk prey items fed one to three times weekly, including the following: domestic crickets and dubia roaches, common anoles and Hawaiian house geckos, tropical fruit mash, dark leafy greens mix, domestic mice (neonate, juvenile or adult), mealworms, rats (juvenile or adult), juvenile domestic chickens, feeder goldfish and rosy red minnows. We scored empirical dietary niche breadth on a scale from 0 to 3 (where 0 represented a single food item fed to animals housed at the EBV, and three represented four food items) to later compare individual isotopic breadth to empirical resource use.

Before preparing specimens for accession into the herpetological collection at the Museum of Vertebrate Zoology at the University of California, Berkeley, individuals were weighed and measured and muscle and liver were dissected for future stable isotopic analysis. These tissues have a twofold difference in isotopic half‐life as recommended by Matich et al. (2021) for studies of individual specialization—muscle has a longer isotopic half‐life than liver across taxa and measures 81 versus 21 days for carbon retention in the lizard *Sceloporus undulatus* (Vander Zanden et al., 2015; Warne et al., 2010). Extra muscle and liver were dissected from each individual at this stage in order to compare stable isotope signatures between delipified and non‐delipified tissues. Additionally, a tail tip was taken from each individual to compare variation in isotopic ratios of whole tail versus pure muscle. Individuals were then fixed in 10% buffered formalin. Specimens remained in the formalin solution until properly fixed into position. This time differed between individuals due to the size of the animal and because of damage to tissues resulting from freezing and thawing of specimens. Once individuals were adequately fixed, specimens were transferred to 70% ethanol. Additional muscle and liver samples were dissected from each specimen at 1, 2, 4 and 8 weeks post transfer.

### 2.2 Stable isotope analysis

Sample preparation and analysis for stable isotope ratios were conducted at the Cornell University Stable Isotope Laboratory. Samples were rinsed of storage buffer, then freeze‐dried and homogenized using a Spex CertiPrep 6750 Freezer/Mill. All samples were delipified using a 2:1 chloroform:methanol extraction procedure (except the duplicate set of fresh tissue samples for comparison with delipified fresh tissue), and all samples were weighed and encapsulated in tin capsules. Carbon and nitrogen stable isotope ratios (δ ^13^C and δ ^15^N respectively) were measured on a Thermo Delta VIRMS coupled to an elemental analyzer (Carlo Erba NC2500). Stable isotope ratios are expressed as delta (δ) in per mil (parts per thousand, ‰) according to the following equation,

(1) $$\left[\frac{R_{\text{sample}}-R_{\text{standard}}}{R_{\text{standard}}}\right]=d_{\text{sample}-\text{standard}}$$

where *R*_sample_ is the ratio of the heavy to light isotope (^13^C/^12^C or ^15^N/^14^N) in the sample, *R*_standard_ is the equivalent ratio in the working reference gas (calibrated against an internationally known IAEA standard, V‐PDB for δ ^13^C and atmospheric nitrogen for δ ^15^N), and *d*_sample‐standard_ is the difference in isotopic composition of the sample relative to the standard, expressed in units per mil. Quality control was performed by comparing the overall standard deviation of the sample runs to an internal animal standard (‘DEER’) and assessing instrument accuracy using a chemical Methionine standard. Isotope corrections were performed using a two‐point normalization (linear regression) of all δ ^13^C and δ ^15^N data using two additional in‐house standards (‘KCRN’—corn and ‘CBT’—trout).

### 2.3 Assessing methodological effects on isotopic differences

#### 2.3.1 Storage buffer

Because ethanol has been shown to have variable effects on carbon stable isotope ratios (Bugoni et al., 2008; Horii et al., 2015; Olin et al., 2014; Shen et al., 2023), all tissues were stored in RNAlater (composed of EDTA—C10H16N2O8, sodium citrate—Na3C6H5O7 and ammonium sulfate—(NH4)2SO4). RNAlater is a salt buffer commonly used to preserve tissues at room temperature for both DNA and RNA downstream analysis, ensuring stability of tissues at room temperature for extended periods of time. As a result, this buffer is ideal for field biologists working in remote locations without access to freezer storage, the ability to transport liquid nitrogen or resources for processing samples in the field.

However, because RNAlater includes several nitrogen‐containing compounds, an additional set of tissues including some collected for this study was subsampled such that half were stored in buffer while the other half were flash‐frozen for comparison. To quantify the effect of salt buffer on stable isotope ratios, additional tissue was taken from 12 specimens throughout the preservation process, as well as from two available specimens not undergoing preservation, and flash‐frozen for comparison with paired samples stored in buffer.

Following stable isotope analysis using the same protocol as above, we first detected and removed outliers using the bagplot() function from the R package aplpack (Wolf, 2019). This function employs a method that generalizes the univariate boxplot to bivariate data, identifying outliers based on the half space location depth of each point in relation to the data as a whole. We first checked for a linear relationship between paired samples before and after buffer storage. We then assessed both univariate and bivariate differences in the two tissue sets using Levene's test for homogeneity of variance, a test for homogeneity of multivariate dispersion and a permutational multivariate analysis of variance (PERMANOVA). The latter method compares variance within groups to variance between groups and is a nonparametric assessment, with the only assumption being that observations are interchangeable under the null hypothesis. Significant differences in the dispersion and centroid of observations are detected by compared test statistics across permutations, wherein values are shuffled between groups. Using the distance matrix calculated from carbon and nitrogen signatures, we used the betadisper() and adonis2() functions in the R package vegan (Oksanen et al., 2025), with observations exchanged among treatments in 999 permutations.

To assess whether bias due to storage effects alters conclusions drawn from downstream analyses, we compared community niche metrics calculated in bivariate isotope space for both groups. The niche metrics used in these calculations include six Layman Metrics that are useful for assessing the structure of variation in isotopic space: (1) δ^15^N range (NR), representing the trophic breadth of the population; (2) δ^13^C range (CR), representing the basal resource diversity utilized by a population; (3) total area of the convex hull (TA) containing C and N isotopic signatures in bivariate space, providing an estimate of the total niche width of a population; (4) mean distance to centroid (CD), another measure of niche width providing information on the dispersion of individuals in niche space; (5) mean nearest neighbour distance (MNND), indicating the degree of density and clustering of individuals in niche space; and (6) standard deviation of the nearest neighbour distance (SDNND), indicating the evenness of spatial density in a population. These metrics were calculated for each genus and subsequently converted into a distance matrix for the control and treatment groups to assess change in ecological structure estimated from bivariate isotope space. We also computed change in individual isotopic breadth before and after storage in buffer using the Euclidean distance between liver and muscle bivariate isotope signatures.

#### 2.3.2 Effects of storage in RNAlater

We found that RNAlater has a strong depleting effect on the δ15N signature of all tissues (Figure S1). Nitrogen signatures do not demonstrate a significant linear relationship among paired samples before and after buffer storage for either liver or muscle (*r* ^2^ = −0.09, *p* = 0.567; *r* ^2^ = −0.267, *p* = 0.115, respectively) (Figure S2). Carbon liver and muscle signatures, however, are virtually unaffected by storage in RNAlater (*r* ^2^ = 0.989, *p* = <2.2 × 10^−16^, *r* ^2^ = 0.899, *p* = 9.3 × 10^−14^, respectively). Subsequently, measures of within‐individual variation based on Euclidean distance between liver and muscle tissue in isotope space before and after buffer storage, as well as Spearman's rank, were not correlated (*r* ^2^ = 0.153, *p* = 0.379; rho = 0.160, *p* = 0.356). Because bivariate measures of within‐individual isotopic breadth from RNAlater‐stored tissue are unreliable, all results below concerning within‐individual variation are based on the univariate difference between carbon isotope signatures of liver and muscle, or Δ^13^C.

However, there was no significant difference in variance of either liver or muscle tissue among individuals with and without RNAlater storage (Levene's test, *p* = 0.81 and 0.86 respectively; *n* = 38 samples across 14 individuals). Homogeneity of dispersion is maintained in bivariate isotope space (*F* = 0.260, *p* = 0.895), but a significant difference was found in centroid location (*F* = 65.226, *R* ^2^ = 0.303, *p* = 0.001). This result is straightforward given that the buffer causes a depletion in nitrogen signatures regardless of tissue. However, community structure based on distances among genera in isotopic niche metrics for both treatment groups is similar (Figures S3 and S4), with effect sizes for each metric three to five orders of magnitude less than one standard deviation. These results suggest that tissues preserved in RNAlater are still meaningful when analysing among‐individual differences, that is niche packing or trophic dispersion analyses. We therefore retain nitrogen signatures for our analyses of community niche structure below. It should also be noted that because carbon isotope ratios remain strongly correlated between flash‐frozen tissue and tissue stored in RNAlater, this storage buffer can be useful for tissue intended solely for carbon stable isotope ratios in addition to genomic and transcriptomic analyses.

#### 2.3.3 Whole tail versus pure muscle

Many field studies of squamate organisms involve sampling the distal tip of the tail as a minimally invasive procedure. Because this yields only a small amount of tissue, especially for small‐bodied lizards and snakes, researchers must often use the entire sample for isotopic analysis rather than dissecting the sample for individual tissue types. However, because existing literature on discrimination factors includes estimates only for specific tissues comprising the tail such as bone, skin and muscle, we aimed to assess whether the isotopic signature of a whole tail sample differed significantly from its individual components. We therefore sampled both whole tail and pure muscle from each individual and compared isotopic ratios using both a t‐test and an *F*‐test to determine univariate equality of variances, as well as PERMANOVA to assess significance of dispersion in bivariate space.

#### 2.3.4 Delipification

Tissues with high lipid content can disrupt the signal of resource use due to the fractionation of carbon during lipid synthesis. However, chemical lipid extraction of tissues prior to stable isotope analysis has also been shown to have an effect on isotope ratios, and existing mathematical corrections are tissue‐ and taxon‐specific (Logan et al., 2008; Sweeting et al., 2006). We therefore aimed to assess whether lipid extraction had a significant effect on stable isotope ratios in squamate muscle and liver tissues. We subdivided muscle and liver tissue from each individual into paired sets such that one subsample was subject to delipification before stable isotope analysis using a 2:1 chloroform:methanol extraction protocol. We compared isotopic ratios using both a t‐test and an *F*‐test to determine univariate equality of variances, as well as PERMANOVA to assess significance of dispersion in bivariate space.

### 2.4 Quantifying effects of fluid preservation on isotopic niche breadth

The absolute value of the difference in carbon isotopic signature of muscle and liver tissues before preservation, Δ^13^C, was used as a proxy for within‐individual isotopic niche breadth and compared to the breadth of diet fed to animals at the East Bay Vivarium. A relatively large Δ^13^C value is interpreted as a more generalized diet compared to a smaller Δ^13^C. Change in individual niche breadth throughout the preservation process was measured as the difference in Δ^13^C between muscle and liver tissues before (Time point 0) and after (Time point 4) preservation.

We assessed differences in the distribution of individuals in niche space before and after preservation using a test for homogeneity of dispersion and PERMANOVA. We additionally delineated two ‘communities’ as groups of genera at Time point 0 and Time point 4 to quantify metrics of dietary niche breadth before and after the preservation process. Layman niche metrics described above, based on variance among individuals, were calculated in bivariate isotopic space using a Bayesian approach (Jackson et al., 2011). Bayesian inference of these metrics is achieved using vague priors to produce a range of probable values for each calculated metric, thus reflecting uncertainty in the estimate (Jackson et al., 2011). Finally, a multivariate model including snout‐vent length (SVL), clade and time spent in formalin was conducted on both muscle and liver tissue to determine whether these factors were related to changes in isotopic signature.

### 2.5 Empirical example

We include an isotopic analysis to explore historical and contemporary aspects of niche variation in *Thamnophis* garter snakes, including a dietary specialist *Thamnophis ordinoides* and a dietary generalist *Thamnophis elegans*. Extensive field studies containing data on stomach contents show that *T. ordinoides* is a slug specialist across its range, while *T. elegans* is a generalist predator consuming fish, anurans and leeches in the geographic region selected for this study (Britt & Bennett, 2008; Kephart & Arnold, 1982; Manier et al., 2007).

Historical samples include 10 *T. ordinoides* and 10 *T. elegans* from the University of California, Berkeley Museum of Vertebrate Zoology herpetological collection. Specimens from each species were selected to represent a single population (collected from the same site or within 10 km), sampled in the same year or as near as chronologically possible (Table S1). Liver and muscle tissues were dissected, freeze‐dried and prepared for isotopic analysis at the Cornell University Stable Isotope Laboratory.

Contemporary samples include 13 individuals of *T. elegans* and 40 individuals of *T. ordinoides* sampled under California Department of Fish and Wildlife Specific Use Permit S‐190150023‐19015‐001 and Institutional Animal Care and Use Committee policy #AUP‐2014‐11‐6857‐1. Individuals of each species were sampled in the same season and same geographic regions as the historical samples (Table S1). Tail tips (<1 cm) were sampled from each individual and stored in RNAlater, and blood was collected opportunistically from the incision site via capillary tube. Blood samples were thawed and dried overnight in encapsulation tins before being processed at the Center for Stable Isotope Biogeochemistry laboratory at University of California, Berkeley. Tail samples were rinsed of buffer using deionized water, freeze‐dried for 24–48 h and homogenized with a MO‐BIO PowerLyzer using reinforced tubes with 3.2‐mm stainless steel beads.

We calculated 95% confidence interval ellipses as well as Bayesian Layman metrics quantifying the temporal dispersion of each population.

## 3 RESULTS

We subsampled tissues with different metabolic turnover rates from a phylogenetically diverse set of squamates before, during and after the standard specimen preservation process to determine whether formalin‐fixed tissues could be used to recover the isotopic dietary niche. Below, we discuss results that will inform methodological decisions for any stable isotope study, as well as broader eco‐evolutionary applications of stable isotope analysis of fluid‐preserved museum specimens.

### 3.1 Isotopic niche breadth and empirical diet breadth

We recovered a positive correlation between carbon isotopic and empirical niche breadths for both lizards (*r* = 0.44, *p* = 0.09) and snakes (*r* = 0.46, *p* = 0.15), suggesting that isotopic differences between muscle and liver do capture a certain degree of variation in resource use. Many factors influence the stable isotope ratios of animal tissue beyond food intake, including disease, dehydration and other physiological conditions (Durso et al., 2020; Reitsema, 2013). A noisy relationship between individual diet and isotopic breadth is therefore expected, but other recent studies demonstrate that multi‐tissue isotopic specialization metrics are robust to varying ranges of source isotope ratios and tissue turnover rates (Arnoldi et al., 2024; Bond et al., 2016).

### 3.2 Effect of lipid extraction

Ratios of percent carbon to nitrogen were <4 for 92% of tissue samples prior to delipification (mean C:N ratio = 1.5, 2.2, and 1.6 for muscle, liver and tail tissues, respectively). These low ratios are in part due to storage in RNAlater, which tended to increase percent nitrogen and decrease percent carbon (Figure S5, Table S1). Delipification further lowered C:N ratios in liver tissue via an increase in percent carbon and decrease in percent nitrogen, but had a minimal effect in muscle tissue (Figure S6). Carbon isotope signatures of both delipified muscle and liver are highly correlated with signatures of paired tissue samples not subjected to delipification (*r* ^2^_muscle_ = 0.77, *p*_muscle_ = 2.20 × 10^−6^; *r* ^2^_liver_ = 0.62, *p*_liver_ = 0.0004) while nitrogen signatures showed a weak relationship for muscle tissue and no relationship for liver (*r* ^2^_muscle_ = 0.38, *p*_muscle_ = 0.05; *r* ^2^_liver_ = 0.13, *p*_liver_ = 0.52) (Figure S7). However, F‐tests to determine equality of variances for both isotopes in both tissue types were nonsignificant. Accordingly, no significant difference is recovered in bivariate dispersion (*F*_muscle_ = 0.0617, *p*_muscle_ = 0.787; *F*_liver_ = 0.260, *p*_liver_ = 0.894) and PERMANOVA analyses (*F*_muscle_ = 0.315, *R* ^2^_muscle_ = 0.006, *p*_muscle_ = 0.681; *F*_liver_ = 0.542, *R* ^2^_liver_ = 0.010, *p*_liver_ = 0.528) when comparing delipified and non‐delipified tissues.

### 3.3 Isotopic signatures of whole tail and pure muscle

Carbon isotope signatures of paired samples of pure muscle and whole tail are not significantly different, as measured by a Welch two sample t‐test (*p* = 0.61) and reflected by a strong positive correlation (*r* = 0.95, *p* = 1.078e^−13^). F‐tests to determine equality of variances were also nonsignificant. Nitrogen signatures of paired muscle and tail samples are significantly differentiated (Welch two sample *t*‐test, *p* = 0.02), though the difference recovered here would not be considered biologically relevant based on average trophic discrimination factors (mean_tail_^15^N = 3.48, mean_muscle_^15^N = 4.66). Further, F‐tests to determine equality of variances of muscle and tail nitrogen ratios are nonsignificant. No significant difference is recovered in bivariate dispersion (*F* = 0.260, *p* = 0.910) and PERMANOVA analyses (*F* = 1.276, *R* ^2^ = 0.023, *p* = 0.267), and Layman metrics based on bivariate isotope signatures are similar for muscle and whole tail ‘communities’ (Figure S8).

### 3.4 Preservation effects on community niche metrics

**FIGURE 1:**
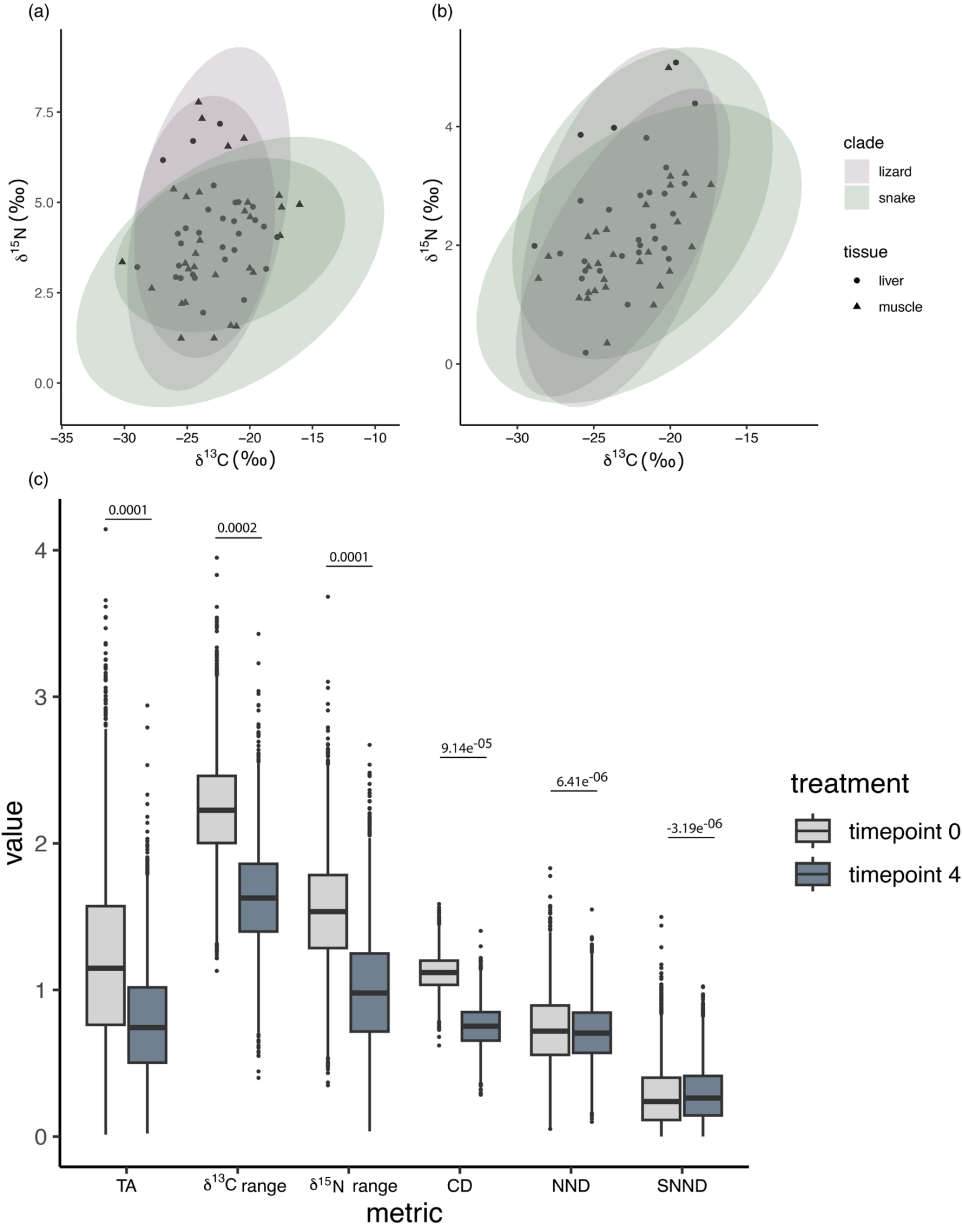
Community niche metrics are not significantly affected by preservation. Standard ellipses before (a) and after (b) specimen preservation. (c) Bayesian Layman metrics before and after specimen preservation. Effect sizes are displayed above each comparison. Preservation results in slightly smaller total niche areas driven by reductions in the range of carbon and nitrogen values, but niche structure represented by dispersion of individuals in isotopic space (nearest‐neighbour distance and associated standard deviation) is unaffected.

We found no significant difference in niche packing as measured by dispersion of individuals in bivariate isotope space following preservation. Homogeneity of dispersion between Time points 0 and 4 was recovered (*F* = 0.378, *p* = 0.54), although the PERMANOVA identified a significant difference in centroid location (*F* = 7.47, *R* ^2^ = 0.060, *p* = 0.004). This is reflected in slight shifts in ellipse orientation that minimize distinction between the two clades (Figure 1a,b). However, the proportion of variance explained is minimal, and we find negligible change between Time 0 and Time 4 in Bayesian estimates of all six Layman metrics measured for lizards and snakes as proxy populations (Figure 1c).

### 3.5 Preservation effects on metrics of individual variation

**FIGURE 2:**
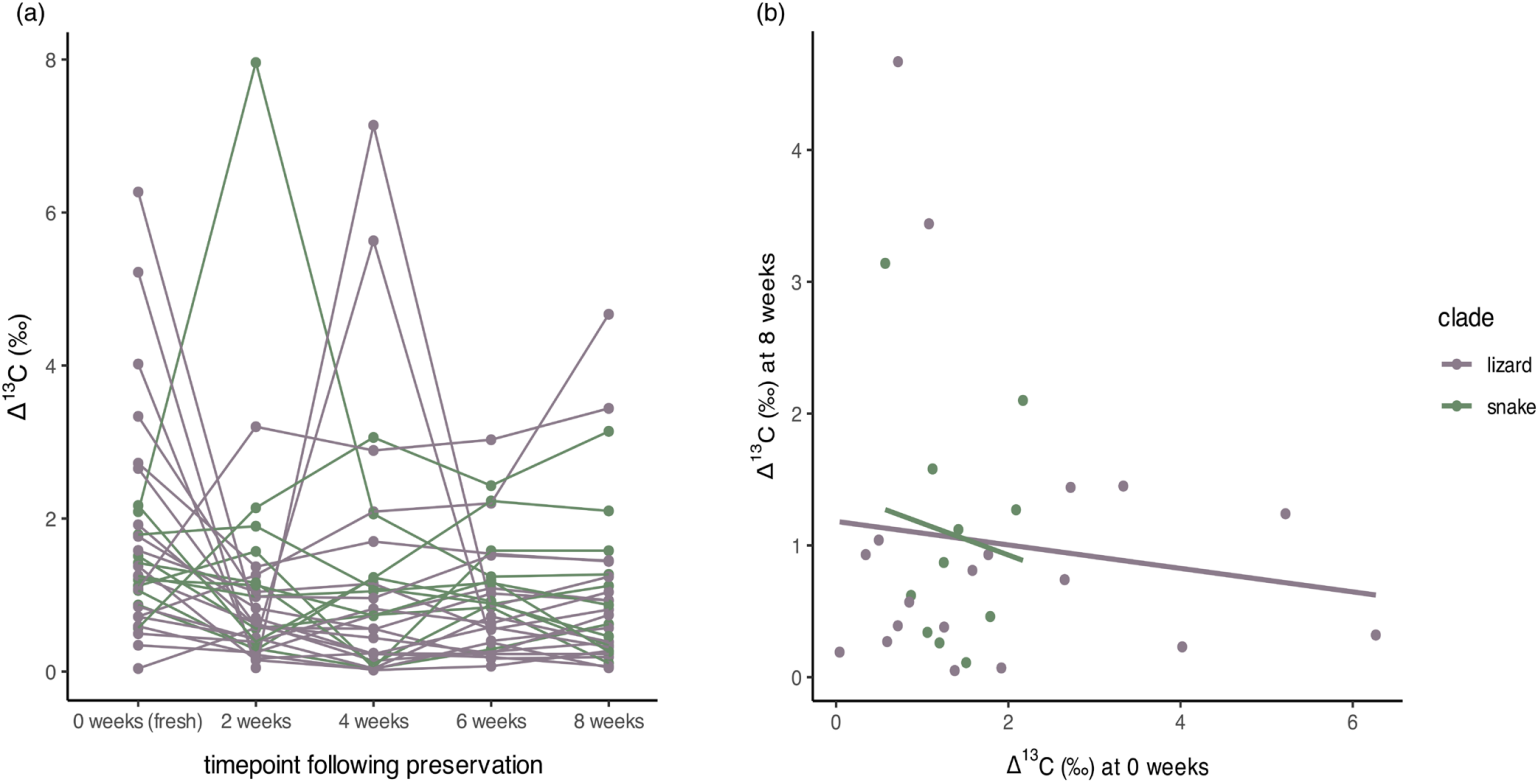
Individual isotopic niche breadth is not recovered following preservation. (a) Within‐individual carbon breadth changes through the preservation process. (b) Individual isotopic breadths before and after preservation are not correlated for lizards. Snakes demonstrate a slight negative correlation related to snout‐vent length, whereby larger specimens show less change in individual isotopic niche breadth following preservation.

Measures of individual isotopic breadth at Time point 0 and Time point 4 are not correlated (*r* = −0.13, *p* = 0.50) (Figures 2a,b), and a rank‐based measure of association also fails to recover a relationship (Spearman's rho = −0.0002, *p* = 0.99), suggesting that the preservation process impedes our ability to recover reliable measures of individual niche width. This is driven by relatively large changes in the carbon isotope signature of muscle in some individuals throughout the preservation process (Figure S9). In a multivariate model including snout‐vent length (SVL), clade, tissue type and time spent in formalin, the total difference in individual isotopic breadth between Time point 0 and Time point 4 is only related to SVL (*r* = −0.30, *p* = 0.1), such that larger individuals experienced smaller discrepancies in isotopic breadth between the start and end of the preservation process.

### 3.6 Contemporary and historical niche metrics in *Thamnophis* garter snakes

**FIGURE 3:**
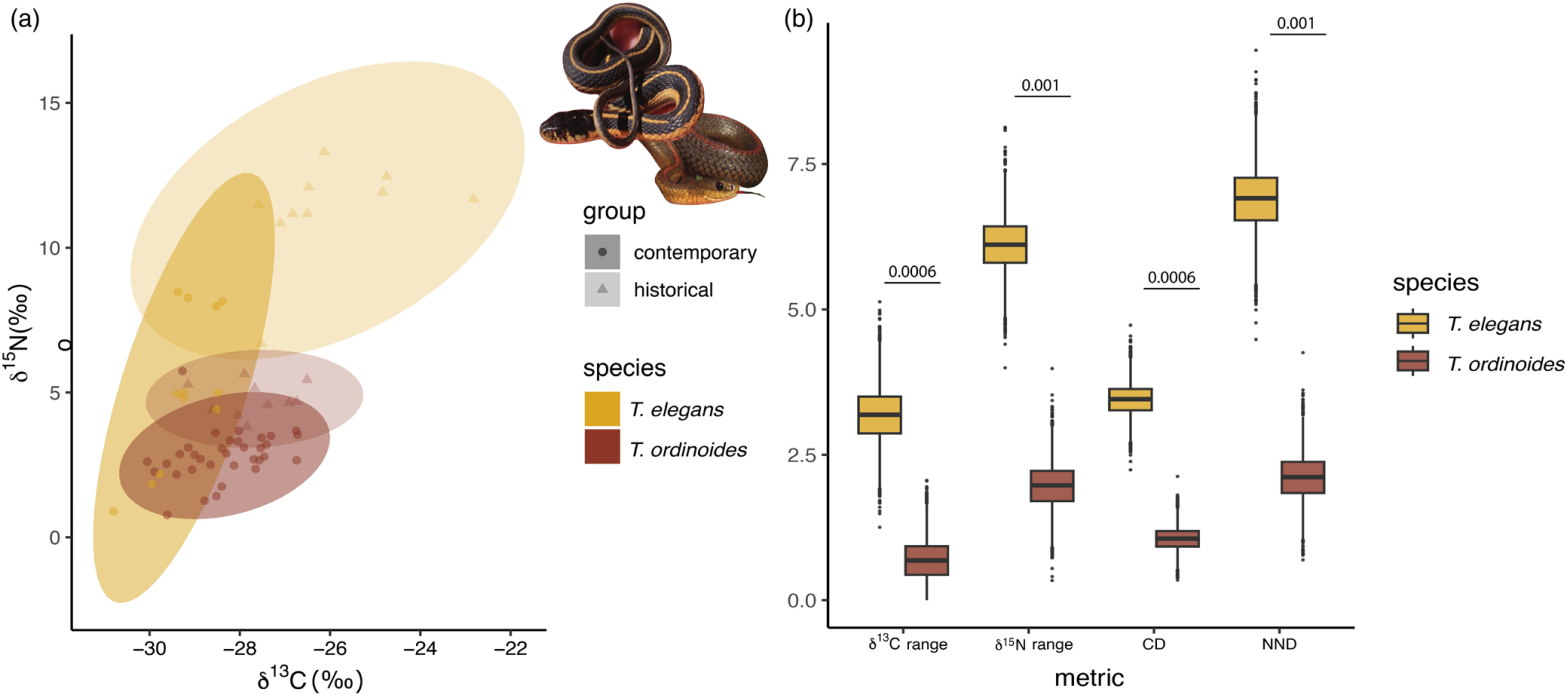
Generalist and specialist populations from the MVZ specimen collection are differentiated in isotopic space. (a) Generalist garter snake *Thamnophis elegans* (yellow) and specialist congener *T. ordinoides* (red) are distinguished in bivariate isotope space, with *T. elegans* occupying more space along both the trophic (nitrogen) and basal resource (carbon) axes. Populations of *T. elegans* also demonstrate temporal variability compared to *T. ordinoides*. (b) Bayesian estimates of niche dispersion between historical and contemporary populations are smaller for *T. ordinoides*, further supporting temporal consistency in resource use. (Photo credit MRG.).

Historical samples of the invertebrate specialist *Thamnophis ordinoides* are clearly distinguished from generalist feeder *T. elegans* in isotopic space (Figure 3b). While the isotopic niches of contemporary and historical *T. ordinoides* appear broadly similar, *T. elegans* demonstrates a contraction in carbon signatures and expansion in nitrogen. Bayesian Layman metrics were calculated that capture dispersion among contemporary and historical groups within each species, with *T. elegans* demonstrating greater temporal variation in all Layman metrics (Figure 3d) except for TA (total area) and SDNND (standard deviation of nearest neighbour distance), which require more groups for accurate calculation. These results demonstrate the useful application of stable isotope analysis in museum specimens to recover more detailed information on historical patterns of niche partitioning.

## 4 DISCUSSION

We conducted a time‐series experiment to determine whether museum specimens can be used to accurately reconstruct characteristics of isotopic niche dispersion, including aspects of within‐ and between‐individual variation. While intraindividual variation could not be reliably recovered for all individuals, other ecologically relevant metrics of niche breadth capturing between‐individual and population variation were not significantly affected by the preservation process. Results from additional tissue comparisons provide useful information for methodological decisions involving stable isotope analysis on field‐collected samples, including a nitrogen depletion effect of RNAlater storage buffer, the isotopic similarities of pure muscle and whole tail, and a negligible effect of lipid extraction. Most importantly, our study demonstrates that with careful consideration, stable isotope analysis of reptile specimens in museum collections can be used to reconstruct community niche dynamics across historical and environmental contexts.

### 4.1 Nitrogen depletion from RNAlater storage buffer

Choosing a method of tissue preservation in field studies is nontrivial when samples demand repeated use for multiple protocols. Prior research has shown that isotopic exchange with chemical preservatives varies among taxa and tissue types (Horii et al., 2015; Sarakinos et al., 2002). Here, we demonstrate a consistent effect of nitrogen depletion across multiple squamate taxa and tissue types that impedes the ability to recover a bivariate metric of individual diet breadth but does not affect dispersion among individuals in bivariate space. Additionally, carbon signatures were highly correlated between flash‐frozen and RNAlater‐preserved tissues, potentially making the salt buffer a better preservative of carbon isotope ratios in squamates. Tissues stored in RNAlater are thus useful for genomic analyses as well as reconstructions of community niche partitioning and basal resource diversity. However, researchers should consider multiple avenues of tissue preservation, should it be accessible to them, to recover the greatest diversity of useful data.

### 4.2 Effects of lipid extraction

In animal tissues, lipids are depleted in ^13^C relative to proteins and carbohydrates, and can therefore contribute to bias in stable isotope ratios when comparing individuals or tissues with varying lipid concentrations (Post et al., 2007). However, the process of removing lipids from tissues before isotope analysis can be timely and expensive. Furthermore, existing studies have shown that when carbon to nitrogen ratios are below 4, indicating low lipid content, carbon isotope values are not significantly affected by the lipid extraction process, and delipification can even cause nitrogen isotope enrichment effects (deVries et al., 2015; Post et al., 2007; Skinner et al., 2016). Our study provides additional evidence that lipid extraction does not significantly affect isotope ratios in both univariate and multivariate dimensions for squamate reptiles, particularly for muscle tissue. However, a caveat to this result is that percent carbon and nitrogen were affected by storage in RNAlater buffer prior to lipid extraction, causing a preliminary decrease in the C:N ratio. Nevertheless, the negligible effect of lipid extraction on the overall dispersion of isotope ratios suggests delipification may not be a necessary step for comparisons of community niche dynamics.

### 4.3 Equivalence of whole tail and muscle

Tail tips are one of the most common tissue types collected in squamate field studies. These samples are often needed for multiple laboratory protocols or may be subsampled repeatedly for museum loans, and tissues may already be very small depending on the size or age of the animal. Dissecting pure muscle from such samples may not yield enough tissue for stable isotope analysis, in addition to being a time‐consuming process. The results from our study comparing isotope ratios from pure muscle vs. whole tail suggest that small squamate tail tips may be used whole for isotopic studies without greatly altering inferred niche metrics. This result is supported by existing research on the carbon retention time of skin and muscle in a small‐bodied lizard, which found a difference of only 13 days between the two tissues (Warne et al., 2010).

### 4.4 Effects of specimen preservation on the isotopic niche

Our study found a significant difference in individual niche breadth calculated from multiple tissues before and after fluid preservation. This result was driven mainly by fluctuations in the carbon signature of muscle tissues, particularly for lizards that experienced unpredictable changes. Because many of the lizard specimens were small‐bodied species or individuals, muscle samples were by necessity taken from different limbs, potentially leading to the observed noise in isotopic signature. Change in individual breadth was moderately related to snout‐vent length in snakes, whereby larger‐bodied specimens demonstrated smaller differences following preservation. This is perhaps due to uneven saturation of the tissue of some larger individuals, suggesting longer experimental timescales and/or replicate sampling of each tissue type at a given time point may be required. Additionally, while stable isotopes represent a record of many feeding events, multiple two‐tissue samples from an individual may be necessary to overcome the noise from other physiological or ecological contributions to isotope ratios (Petta et al., 2020).

In contrast, we recovered no significant variation in the multivariate dispersion of individuals in isotopic space before and after fluid specimen preservation. This important result suggests that studies of niche dynamics can be conducted using museum specimens without the need to rely on preserved stomach contents. This is especially valuable for animals such as snakes that are rarely collected with stomach contents. For contemporary populations that have historical specimen representatives, this result presents an opportunity to compare current ecological dynamics with past trophic patterns.

Concordantly, our comparison of niche structure between historical and contemporary populations of *Thamnophis* garter snakes demonstrates patterns of both niche continuity and dynamism that merit further exploration in a multivariate framework. Such comparisons could prove especially useful in instances of habitat change or introductions of non‐indigenous species, allowing researchers to assess the ecological impacts of such events along a broader temporal axis and at finer scales of niche subdivision.

It is important to note that because variation in carbon and nitrogen isotope ratios may be driven by an organism's physiological state in addition to diet, habitat and environmental variation, caution should be exercised when extrapolating characteristics of the isotopic niche to macroecological scales. Some research questions may be better served by analysing isotopic ratios from inert tissues (hair, feathers, claws, scutes, etc.) which are not subject to metabolic turnover and thus function as ‘snapshots’ in time when the tissue was first synthesized.

The integration of stable isotope analysis with museum collections has recently been called for in freshwater research, with the goal of creating an ‘extended specimen network’ whereby diverse individual data are leveraged to make inferences regarding community and ecosystem changes (Turner et al., 2023). The results of this study suggest similar methods should be applied to squamate specimens, with further investigation directed at individual and tissue‐specific changes in isotope ratios during the preservation process.

## AUTHOR CONTRIBUTIONS

Maggie R. Grundler designed the study, conducted lab work, analysed data and wrote the manuscript. Erica Bree Rosenblum provided funding and logistical support and contributed critically to the manuscript.

## CONFLICT OF INTEREST STATEMENT

The authors have no conflicts of interest to declare.

## STATEMENT ON INCLUSION

This was an experimental study on data collection using museum specimens sourced from and located in the United States. As such, there was no local field work or global distribution of data involving other communities or necessitating regional expertise.

## Supporting information


**Figure S1.** Storage in RNAlater buffer depletes nitrogen signatures regardless of tissue type.
**Figure S2.** Nitrogen signatures of liver and muscle tissue following storage in RNAlater buffer are not significantly correlated with true nitrogen signatures (liver: *r* ^2^ = −0.09, *p* = 0.567; muscle: *r* ^2^ = −0.267, *p* = 0.115).
**Figure S3.** Storage in RNAlater does not change qualitative results of community niche dispersion.
**Figure S4.** Community niche metrics are not significantly affected by storage in salt buffer.
**Figure S5.** Storage in RNAlater (A) reduces percent carbon and (B) increases percent nitrogen regardless of tissue type, resulting in (C) a decrease of C:N ratios.
**Figure S6.** Lipid extraction (A) increases percent nitrogen and (B) reduces percent carbon in liver tissue resulting in (C) a decrease of C:N ratios in liver. Delipification has little effect on percent carbon and nitrogen of muscle tissue.
**Figure S7.** (A) Carbon signatures remain highly correlated to true values following lipid extraction of tissue (*r* ^2^_muscle_ = 0.77, *p*_muscle_ = 2.20 × 10^−6^; *r* ^2^_liver_ = 0.62, *p*_liver_ = 0.0004) while (B) nitrogen signatures do not (*r* ^2^_muscle_ = 0.38, *p*_muscle_ = 0.05; *r* ^2^_liver_ = 0.13, *p*_liver_ = 0.52). The latter are affected by storage in RNAlater buffer for this study.
**Figure S8.** Community niche metrics based on muscle and whole tail tissue. Layman metrics are similar for paired muscle and tail tissues.
**Figure S9.** Change in carbon over time by individual.
**Table S1.** Contemporary tissue samples and historical museum specimens used for comparison of specialist and generalist *Thamnophis* garter snake dietary niches over time (Figure 3, main text).
**Table S2.** Percent carbon and nitrogen from tissues flash frozen compared to storage in RNAlater buffer. RNAlater buffer decreases percent carbon and increases percent nitrogen in all tissues.

## Data Availability

Data available from the Dryad Digital Repository: https://doi.org/10.5061/dryad.k98sf7mmr (Grundler & Rosenblum, 2025).
