# Supplementary material for: Preservation of stable isotope niche dynamics in squamate museum specimens
Source: J Anim Ecol. 2026 Jan 19;95(3):509–20. doi: 10.1111/1365-2656.70212 (PMC12957730; doi:10.1111/1365-2656.70212)
Supplement: Supplementary file 1 — Figure S1. Storage in RNAlater buffer depletes nitrogen signatures regardless of tissue type. Figure S2. Nitrogen signatures of liver and muscle tissue following storage in RNAlater buffer are not significantly correlated with true nitrogen signatures (liver: r 2 = −0.09, p = 0.567; muscle: r 2 = −0.267, p = 0.115). Figure S3. Storage in RNAlater does not change qualitative results of community niche dispersion. Figure S4. Community niche metrics are not significantly affected by storage in salt buffer. Figure S5. Storage in RNAlater (A) reduces percent carbon and (B) increases percent nitrogen regardless of tissue type, resulting in (C) a decrease of C:N ratios. Figure S6. Lipid extraction (A) increases percent nitrogen and (B) reduces percent carbon in liver tissue resulting in (C) a decrease of C:N ratios in liver. Delipification has little effect on percent carbon and nitrogen of muscle tissue. Figure S7. (A) Carbon signatures remain highly correlated to true values following lipid extraction of tissue (r 2 muscle = 0.77, p muscle = 2.20 × 10^−6^; r 2 liver = 0.62, p liver = 0.0004) while (B) nitrogen signatures do not (r 2 muscle = 0.38, p muscle = 0.05; r 2 liver = 0.13, p liver = 0.52). The latter are affected by storage in RNAlater buffer for this study. Figure S8. Community niche metrics based on muscle and whole tail tissue. Layman metrics are similar for paired muscle and tail tissues. Figure S9. Change in carbon over time by individual. Table S1. Contemporary tissue samples and historical museum specimens used for comparison of specialist and generalist Thamnophis garter snake dietary niches over time (Figure 3, main text). Table S2. Percent carbon and nitrogen from tissues flash frozen compared to storage in RNAlater buffer. RNAlater buffer decreases percent carbon and increases percent nitrogen in all tissues. [file JANE-95-509-s001.docx]

SUPPLEMENTARY MATERIALS


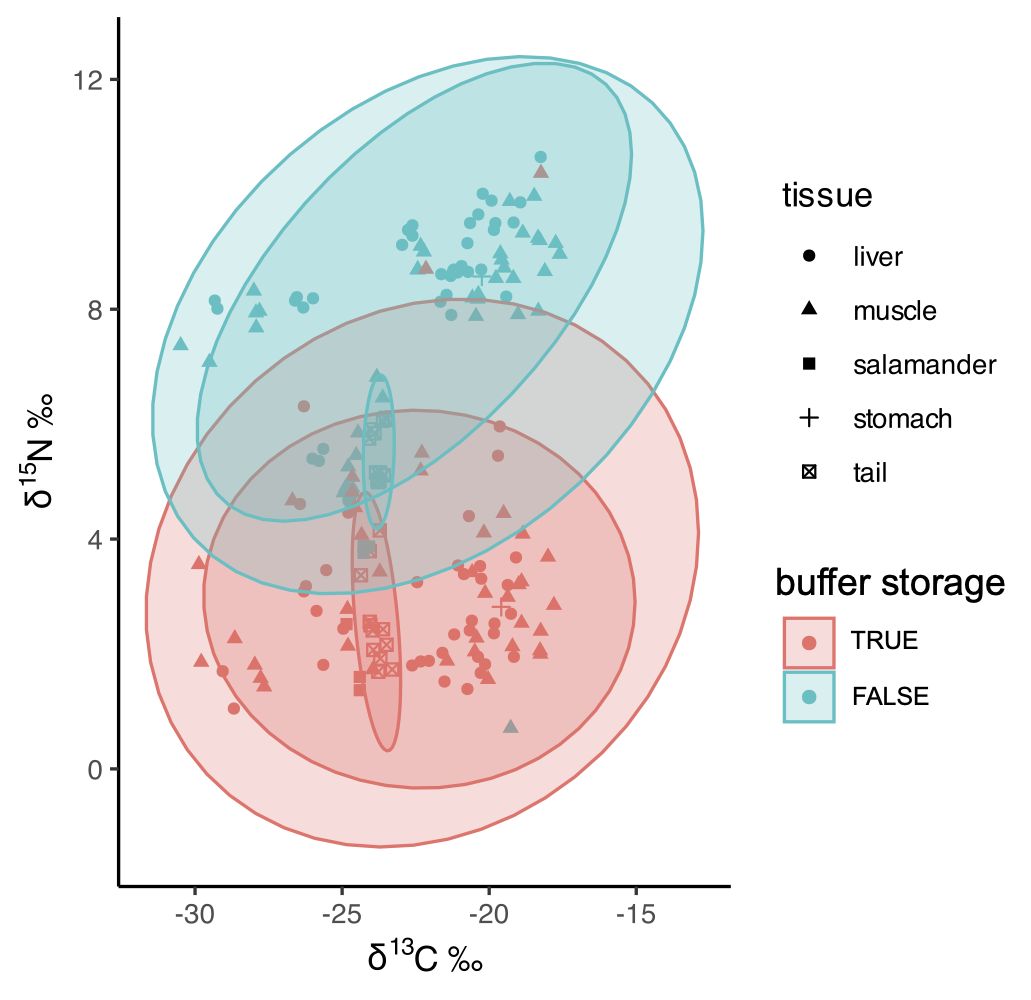


**Figure S1.** Storage in RNAlater buffer depletes nitrogen signatures regardless of tissue type.


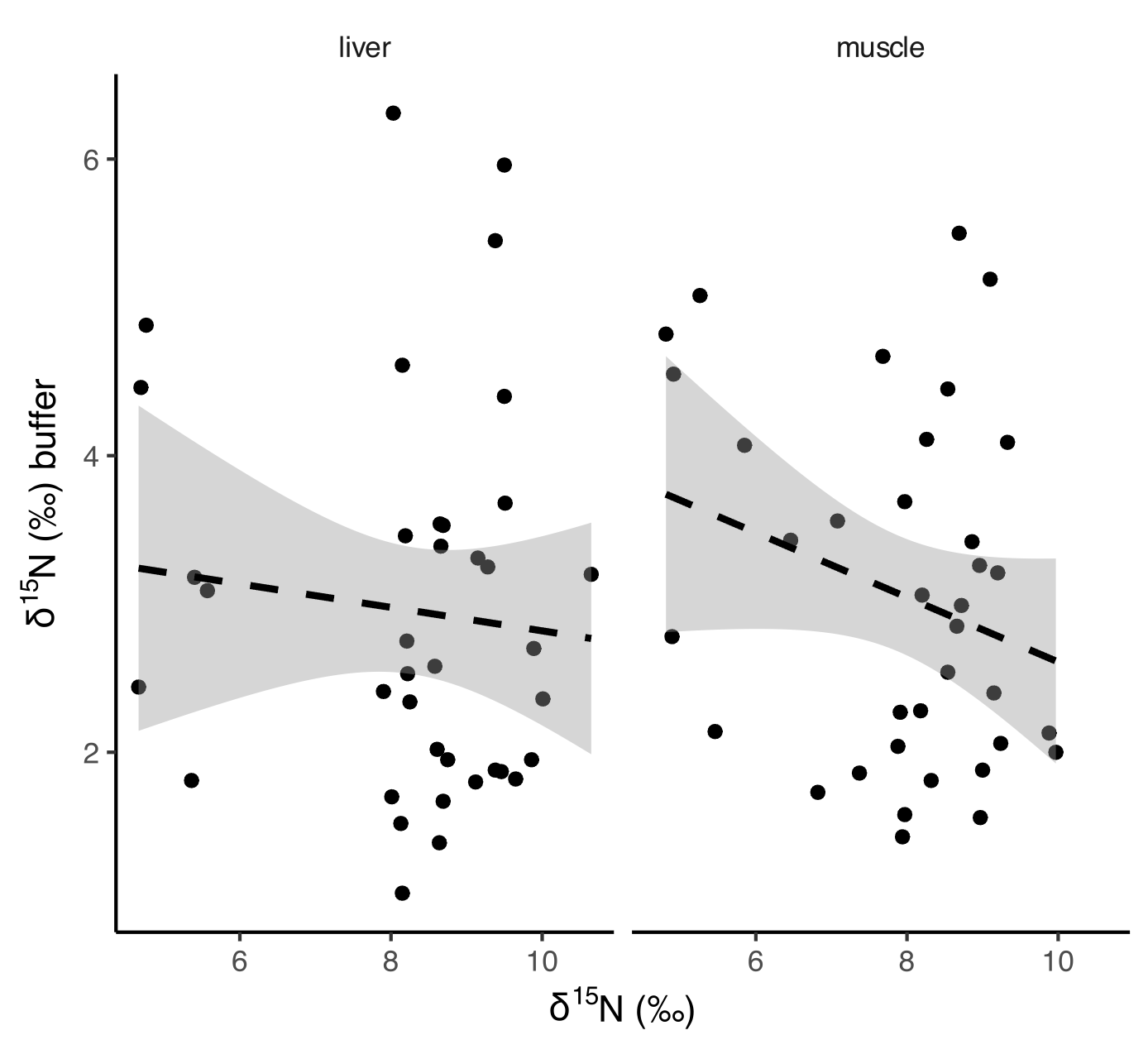


**Figure S2.** Nitrogen signatures of liver and muscle tissue following storage in RNAlater buffer are not significantly correlated with true nitrogen signatures (liver: r^2^ = -0.09, p = 0.567; muscle: r^2^ = -0.267, p = 0.115).


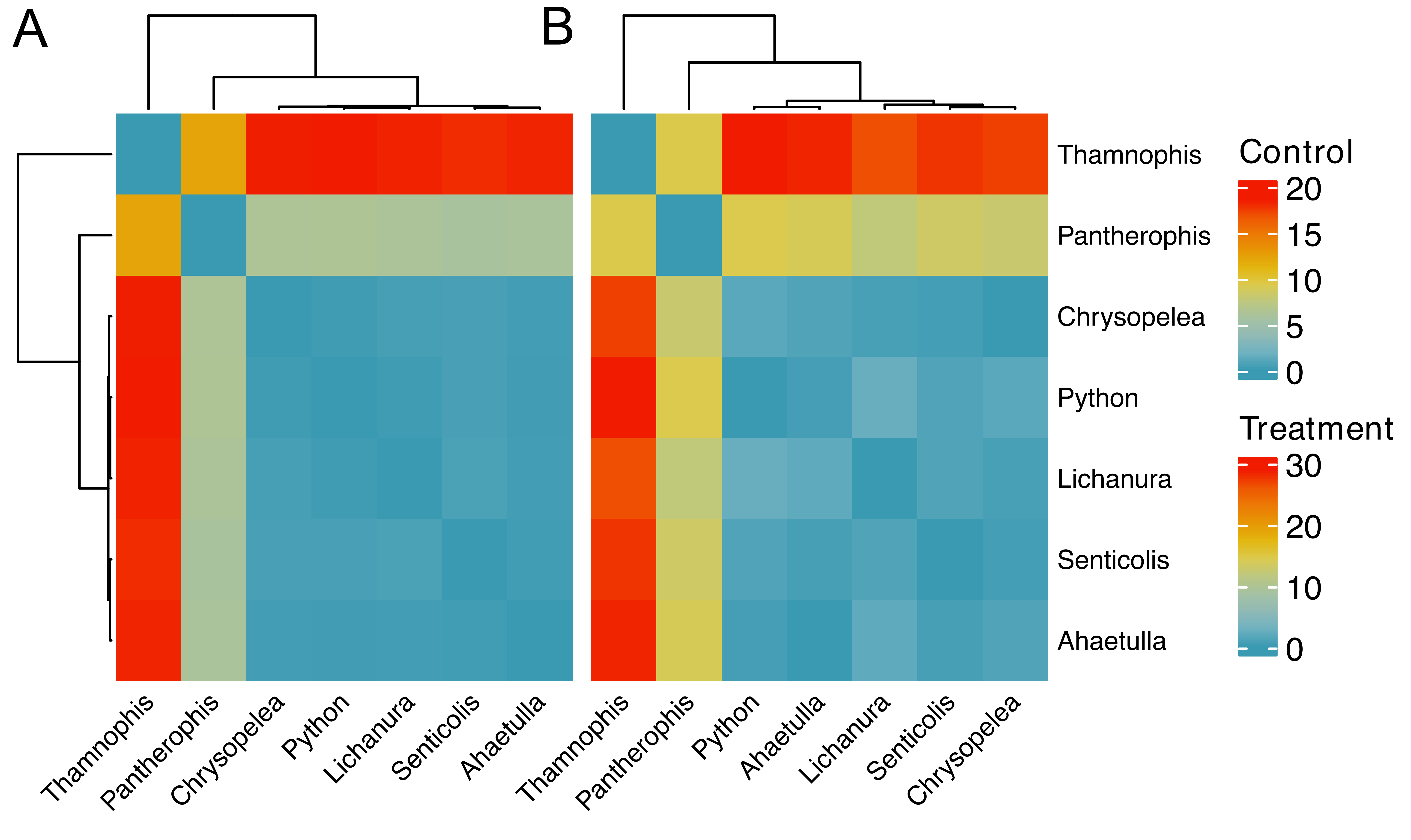


**Figure S3. Storage in RNAlater does not change qualitative results of community niche dispersion.** Heatmaps represent distances between genera in Layman niche metrics for (A) flash frozen tissue and (B) tissue stored in RNAlater. Major clade structure remains unchanged. Species in the larger clade are shuffled, but dendrogram height distance suggests no significant difference in distance between these species both before and after buffer storage.

**
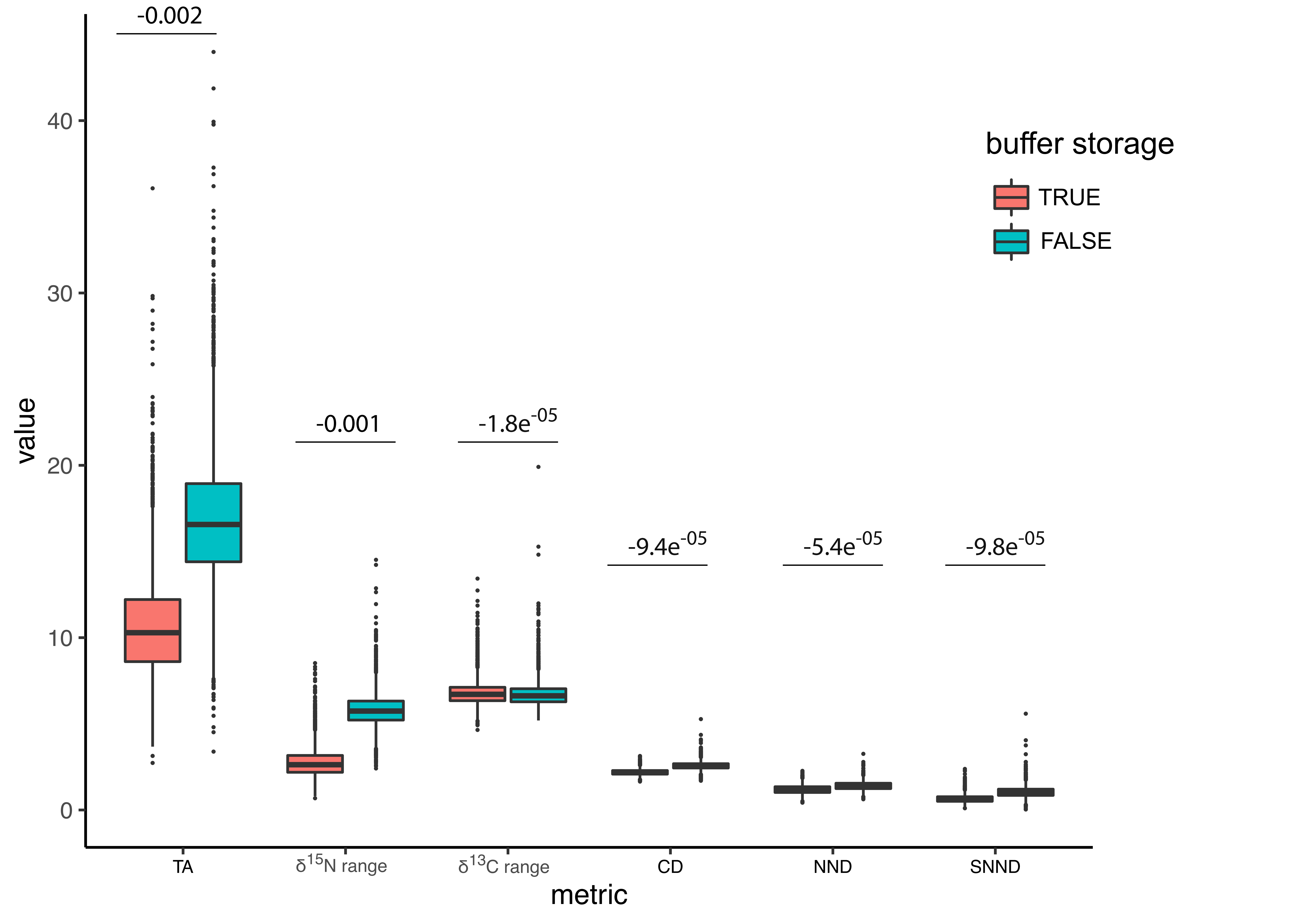
**

**Figure S4. Community niche metrics are not significantly affected by storage in salt buffer.** Bayesian Layman metrics for paired tissues with and without storage in RNAlater. Effect sizes are displayed above each comparison.

| **Species** | **MVZ Catalog Number** | **Date Captured** | **Locality** |
| --- | --- | --- | --- |
| *T. ordinoides* | *N/A* | 21-Aug-19 | Redwood National Park, CA |
| *T. ordinoides* | *N/A* | 31-May-20 | Redwood National Park, CA |
| *T. ordinoides* | *N/A* | 31-May-20 | Redwood National Park, CA |
| *T. ordinoides* | *N/A* | 1-Jun-20 | Redwood National Park, CA |
| *T. ordinoides* | *N/A* | 2-Jun-20 | Redwood National Park, CA |
| *T. ordinoides* | *N/A* | 2-Jun-20 | Redwood National Park, CA |
| *T. ordinoides* | *N/A* | 4-Jun-20 | Redwood National Park, CA |
| *T. ordinoides* | *N/A* | 4-Jun-20 | Redwood National Park, CA |
| *T. ordinoides* | *N/A* | 5-Jun-20 | Redwood National Park, CA |
| *T. ordinoides* | *N/A* | 5-Jun-20 | Redwood National Park, CA |
| *T. ordinoides* | *N/A* | 5-Jun-20 | Redwood National Park, CA |
| *T. ordinoides* | *N/A* | 5-Jun-20 | Redwood National Park, CA |
| *T. ordinoides* | *N/A* | 5-Jun-20 | Redwood National Park, CA |
| *T. ordinoides* | *N/A* | 5-Jun-20 | Redwood National Park, CA |
| *T. ordinoides* | *N/A* | 5-Jun-20 | Redwood National Park, CA |
| *T. ordinoides* | *N/A* | 5-Jun-20 | Redwood National Park, CA |
| *T. ordinoides* | *N/A* | 5-Jun-20 | Redwood National Park, CA |
| *T. ordinoides* | *N/A* | 5-Jun-20 | Redwood National Park, CA |
| *T. ordinoides* | *N/A* | 5-Jun-20 | Redwood National Park, CA |
| *T. ordinoides* | *N/A* | 26-Aug-20 | Redwood National Park, CA |
| *T. ordinoides* | *N/A* | 26-Aug-20 | Redwood National Park, CA |
| *T. ordinoides* | *N/A* | 26-Aug-20 | Redwood National Park, CA |
| *T. ordinoides* | *N/A* | 26-Aug-20 | Redwood National Park, CA |
| *T. ordinoides* | *N/A* | 26-Aug-20 | Redwood National Park, CA |
| *T. ordinoides* | *N/A* | 27-Aug-20 | Redwood National Park, CA |
| *T. ordinoides* | *N/A* | 7-May-21 | Redwood National Park, CA |
| *T. ordinoides* | *N/A* | 8-May-21 | Redwood National Park, CA |
| *T. ordinoides* | *N/A* | 4-Sep-21 | Redwood National Park, CA |
| *T. ordinoides* | *N/A* | 4-Sep-21 | Redwood National Park, CA |
| *T. ordinoides* | *N/A* | 4-Sep-21 | Redwood National Park, CA |
| *T. ordinoides* | *N/A* | 4-Sep-21 | Redwood National Park, CA |
| *T. ordinoides* | *N/A* | 4-Sep-21 | Redwood National Park, CA |
| *T. ordinoides* | *N/A* | 4-Sep-21 | Redwood National Park, CA |
| *T. ordinoides* | *N/A* | 4-Sep-21 | Redwood National Park, CA |
| *T. elegans* | *N/A* | 19-Jun-22 | Sagehen Creek Field Station, CA |
| *T. elegans* | *N/A* | 20-Jun-22 | Sagehen Creek Field Station, CA |
| *T. elegans* | *N/A* | 20-Jun-22 | Sagehen Creek Field Station, CA |
| *T. elegans* | *N/A* | 20-Jun-22 | Sagehen Creek Field Station, CA |
| *T. elegans* | *N/A* | 20-Jun-22 | Sagehen Creek Field Station, CA |
| *T. elegans* | *N/A* | 21-Jun-22 | Sagehen Creek Field Station, CA |
| *T. elegans* | *N/A* | 19-Aug-23 | Kyburz Flat, CA |
| *T. elegans* | *N/A* | 19-Aug-23 | Kyburz Flat, CA |
| *T. elegans* | *N/A* | 19-Aug-23 | Kyburz Flat, CA |
| *T. elegans* | *N/A* | 19-Aug-23 | Kyburz Flat, CA |
| *T. elegans* | *N/A* | 19-Aug-23 | Kyburz Flat, CA |
| *T. elegans* | *N/A* | 19-Aug-23 | Kyburz Flat, CA |
| *T. ordinoides* | MVZ:Herp:17551 | 12-Jul-34 | Klamath, CA |
| *T. ordinoides* | MVZ:Herp:18128 | 12-Jul-34 | Klamath, CA |
| *T. ordinoides* | MVZ:Herp:18213 | 3-Jun-35 | Klamath, CA |
| *T. ordinoides* | MVZ:Herp:18214 | 10-Jun-35 | Klamath, CA |
| *T. ordinoides* | MVZ:Herp:18217 | 9-Jun-35 | Klamath, CA |
| *T. ordinoides* | MVZ:Herp:18218 | 9-Jun-35 | Klamath, CA |
| *T. ordinoides* | MVZ:Herp:18394 | 16-Jun-35 | Smith River, CA |
| *T. ordinoides* | MVZ:Herp:47776 | 12-Aug-47 | Requa, CA |
| *T. ordinoides* | MVZ:Herp:47782 | 12-Aug-47 | Requa, CA |
| *T. ordinoides* | MVZ:Herp:47792 | 12-Aug-47 | Requa, CA |
| *T. elegans* | MVZ:Herp:51724 | Jun-48 | Eagle Lake, CA |
| *T. elegans* | MVZ:Herp:51725 | 14-Jun-48 | Eagle Lake, CA |
| *T. elegans* | MVZ:Herp:51727 | 15-Jun-48 | Eagle Lake, CA |
| *T. elegans* | MVZ:Herp:51728 | 15-Jun-48 | Eagle Lake, CA |
| *T. elegans* | MVZ:Herp:51729 | 15-Jun-48 | Eagle Lake, CA |
| *T. elegans* | MVZ:Herp:51730 | 16-Jun-48 | Eagle Lake, CA |
| *T. elegans* | MVZ:Herp:51731 | 20-Jun-48 | Eagle Lake, CA |
| *T. elegans* | MVZ:Herp:51732 | 22-Jun-48 | Eagle Lake, CA |
| *T. elegans* | MVZ:Herp:51733 | 24-Jun-48 | Eagle Lake, CA |
| *T. elegans* | MVZ:Herp:51734 | 29-Jun-48 | Eagle Lake, CA |

**Table S1.** Contemporary tissue samples and historical museum specimens used for comparison of specialist and generalist *Thamnophis* garter snake dietary niches over time (Figure 3, main text).


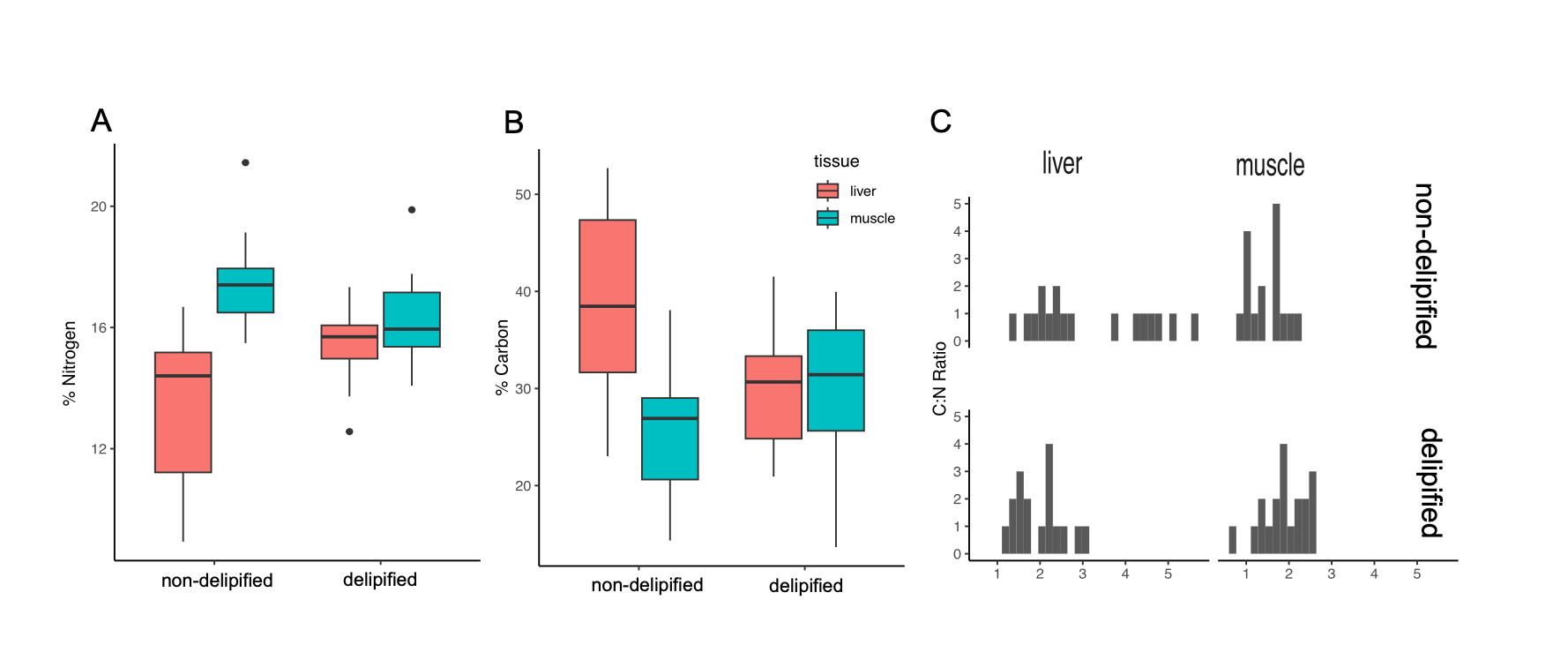


**Figure S5.** Storage in RNAlater (A) reduces percent carbon and (B) increases percent nitrogen regardless of tissue type, resulting in (C) a decrease of C:N ratios

**Fig**
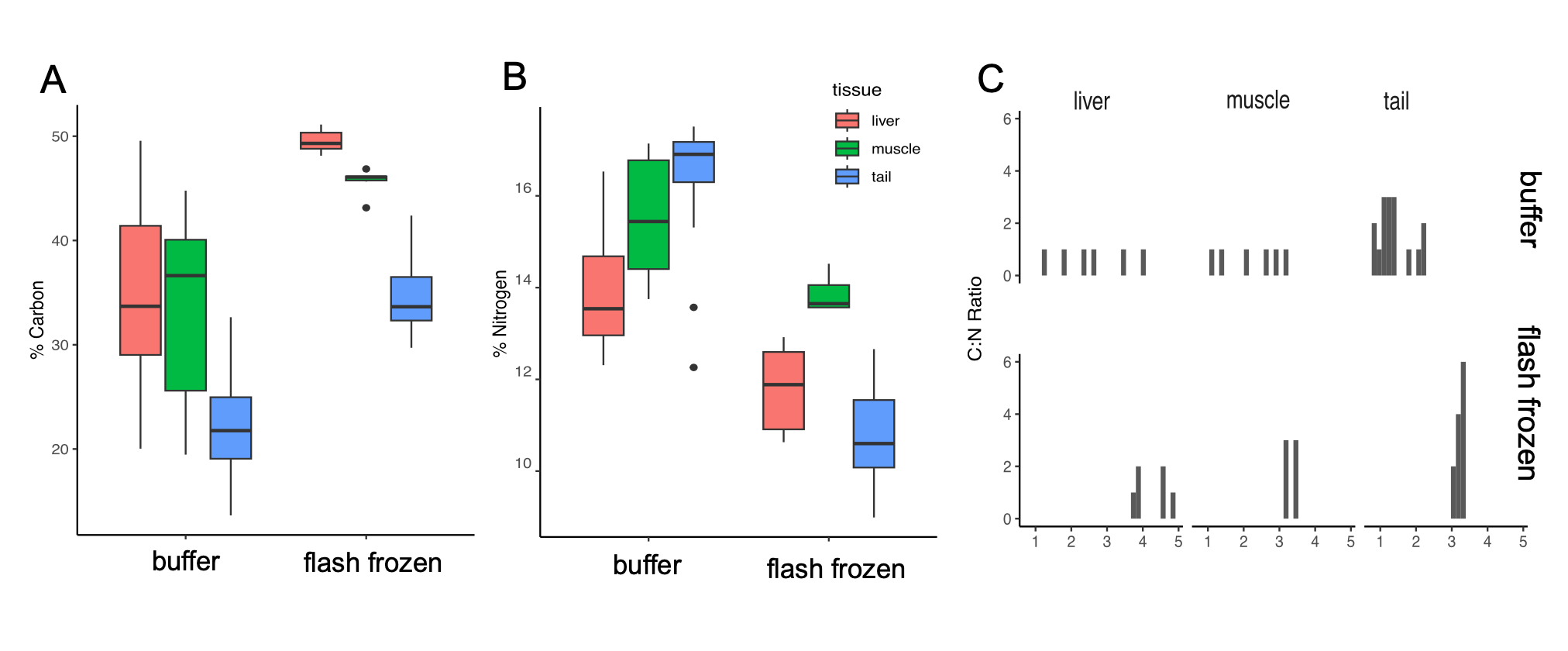
**ure S6.** Lipid extraction (A) increases percent nitrogen and (B) reduces percent carbon in liver tissue resulting in (C) a decrease of C:N ratios in liver. Delipification has little effect on percent carbon and nitrogen of muscle tissue.

| **Tissue** | **Mean %C _flash frozen_** | **Mean % C_buffer_** | **Mean %N_flash frozen_** | **Mean %N_buffer_** |
| --- | --- | --- | --- | --- |
| *liver* | 49.53 | 34.74 | 11.79 | 13.96 |
| *muscle* | 45.66 | 33.44 | 13.85 | 15.51 |
| *whole tail* | 34.89 | 21.78 | 10.78 | 16.47 |

**Table S2.** Percent carbon and nitrogen from tissues flash frozen compared to storage in RNAlater buffer. RNAlater buffer decreases percent carbon and increases percent nitrogen in all tissues.


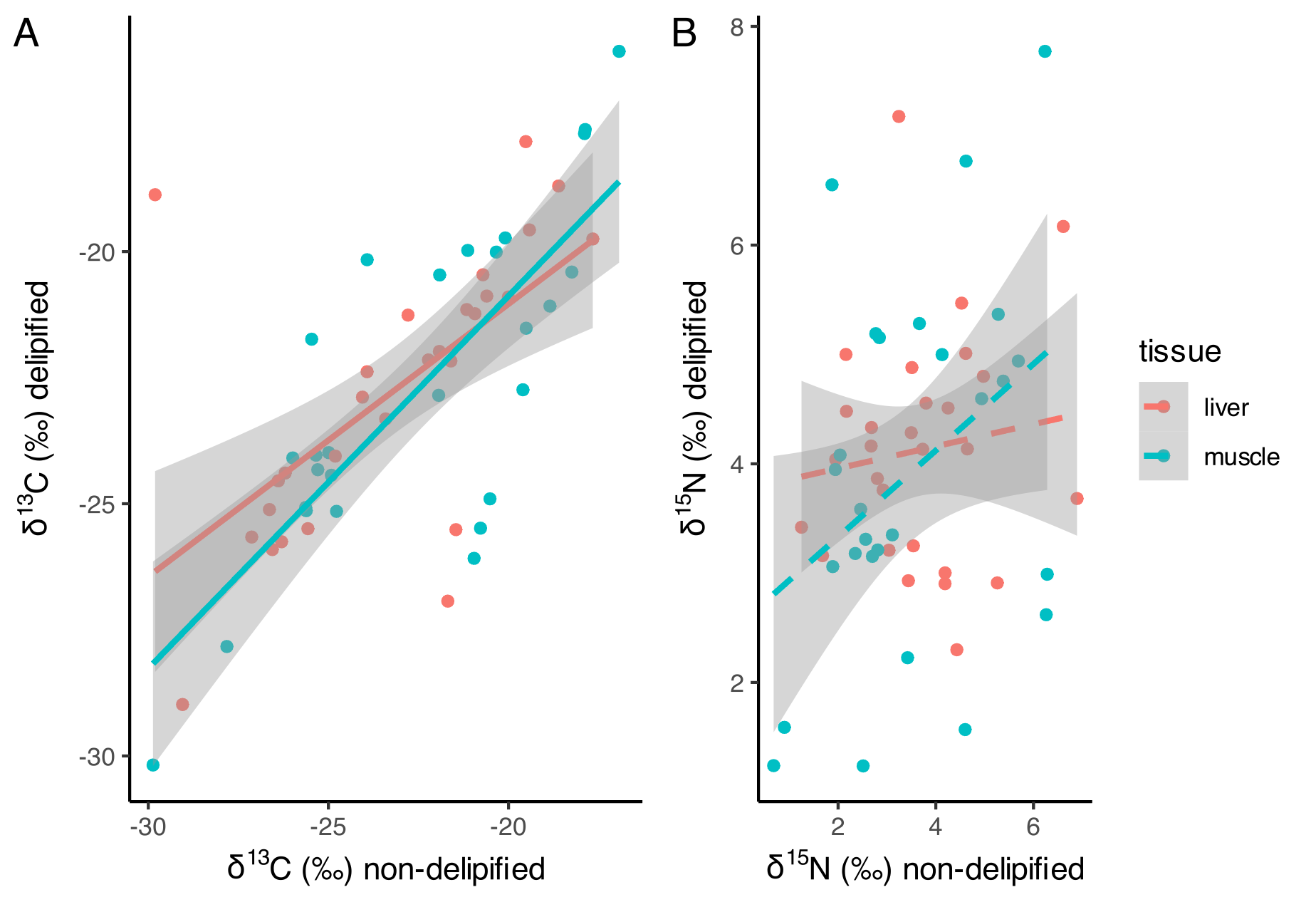


**Figure S7.** (A) Carbon signatures remain highly correlated to true values following lipid extraction of tissue (r^2^_muscle_ = 0.77, p_muscle_ = 2.20 x 10^-6^; r^2^_liver_ = 0.62, p_liver_ = 0.0004) while (B) nitrogen signatures do not (r^2^_muscle_ = 0.38, p_muscle_ = 0.05; r^2^_liver_ = 0.13, p_liver_ = 0.52). The latter are affected by storage in RNAlater buffer for this study.

**Figure S8. *Community niche metrics based on muscle and whole tail tissue.*** Layman metrics are similar for paired muscle and tail tissues.


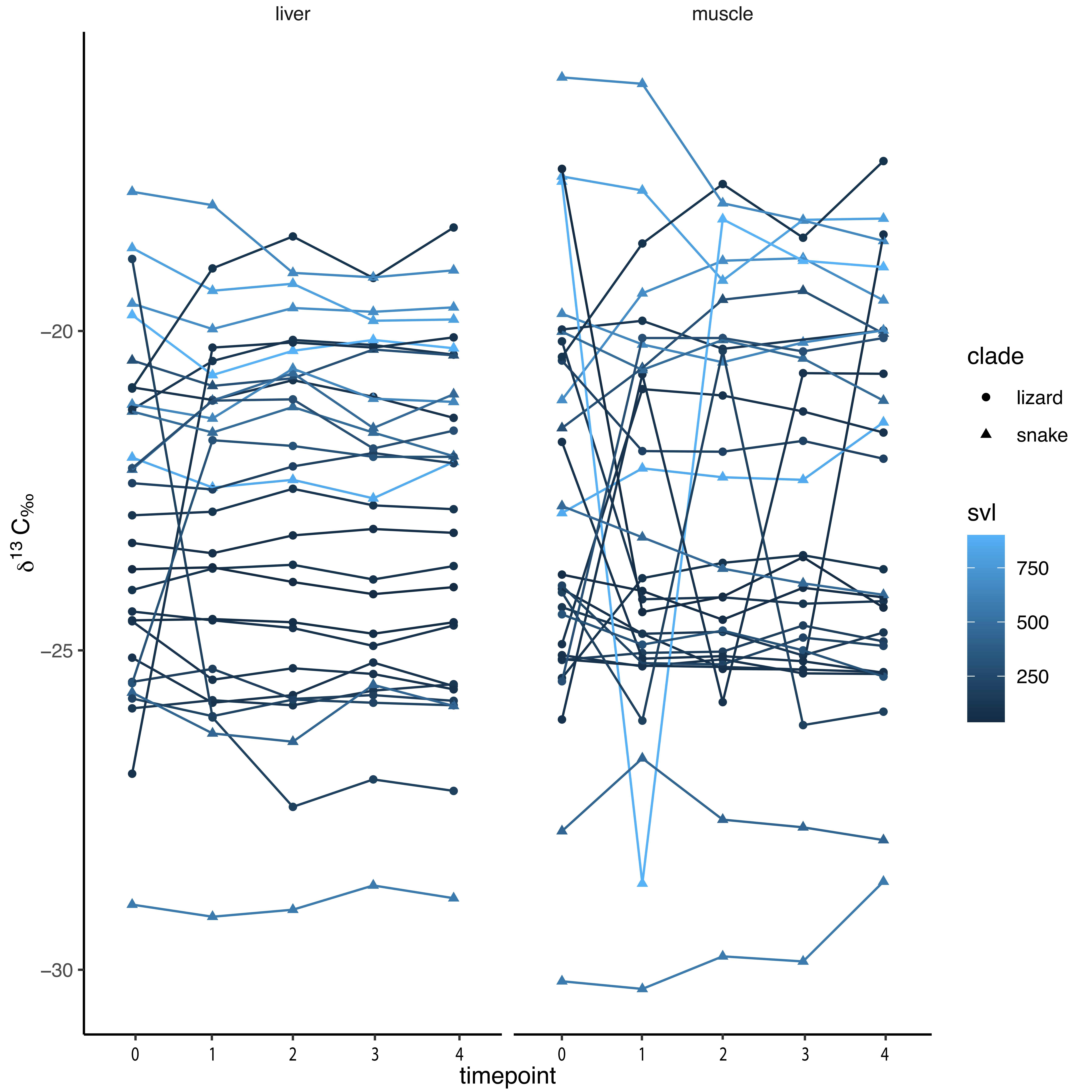


**Figure S9. Change in carbon over time by individual.** Muscle tissue shows greater deviations in carbon signature throughout the preservation process, possibly due to differences in saturation of fixation and preservation fluids.
